# Nationwide continuous monitoring of end-of-life care via representative networks of general practitioners in Europe

**DOI:** 10.1186/1471-2296-14-73

**Published:** 2013-06-03

**Authors:** Lieve Van den Block, Bregje Onwuteaka-Philipsen, Koen Meeussen, Gé Donker, Francesco Giusti, Guido Miccinesi, Viviane Van Casteren, Tomas Vega Alonso, Oscar Zurriaga, Luc Deliens

**Affiliations:** 1 End-of-Life Care Research Group, Vrije Universiteit Brussel and Ghent University, Laarbeeklaan 103, Brussels 1090, Belgium; 2 Department of Public and Occupational Health, EMGO Institute for Health and Care Research, VU University Medical Center, Van der Boechorststraat 7, Amsterdam, BT 1081, The Netherlands; 3 Dutch Sentinel General Practice Network, NIVEL, Otterstraat 118-124, Utrecht, CR 3513, The Netherlands; 4 Cancer Prevention and Research Institute, ISPO, Via Oblate 2,Pal 28/A, Florence 50142, Italy; 5 Scientific Institute of Public Health, J. Wytsmanstraat 14, Brussels 1050, Belgium; 6 Public Health Directorate, Junta de Castilla y León, Paseo Zorilla 1, Valladolid 47071, Spain; 7 Dirección General de Salud Pública, Conselleria de Sanidad, Valencia, Spain; 8 Spanish Consortium for Research in Epidemiology and Public Health CIBERESP, Madrid, Spain

**Keywords:** Palliative care, Monitoring, End of life

## Abstract

**Background:**

Although end-of-life care has become an issue of great clinical and public health concern in Europe and beyond, we lack population-based nationwide data that monitor and compare the circumstances of dying and care received in the final months of life in different countries. The European Sentinel GP Networks Monitoring End of Life Care (EURO SENTIMELC) study was designed to describe and compare the last months of life of patients dying in different European countries. We aim to describe how representative GP networks in the EURO SENTIMELC study operate to monitor end of life care in a country, to describe used methodology, research procedures, representativity and characteristics of the population reached using this methodology.

**Methods:**

Nationwide representative Networks of General Practitioners (GPs) – ie epidemiological surveillance systems representative of all GPs in a country or large region of a country – in Belgium, the Netherlands, Italy and Spain continuously registered every deceased patient (>18 year) in their practice, using weekly standardized registration forms, during two consecutive years (2009–2010).

All GPs were asked to identify patients who had died “non-suddenly”. The last three months of these patients’ lives was surveyed retrospectively. Several quality control measures were used to ensure data of high scientific quality.

**Results:**

A total of 6858 deaths were registered of which two thirds died non-suddenly (from 62% in the Netherlands to 69% in Spain), representative for the GP populations in the participating countries. Of all non-sudden deaths, between 32% and 44% of deaths were aged 85 or older; between 46% and 54% were female, and between 23% and 49% died at home. Cancer was cause of death in 37% to 53% of non-sudden death cases in the four participating countries.

**Conclusion:**

Via the EURO SENTI-MELC methodology, we can build a descriptive epidemiological database on end-of-life care provision in several EU countries, measuring across setting and diseases. The data can serve as baseline measurement to compare and monitor end-of-life care over time. The use of representative GP networks for end-of-life care monitoring has huge potential in Europe where several of these networks are operational.

## Background

How people die and the care delivered in the final phase of life has become an issue of great clinical and public health importance [1,2]. Nevertheless, population-based and nationwide research monitoring the circumstances surrounding death and the quality of the end-of-life care provided in the final months of life is limited [3-5]. Existing empirical research is often restricted to specific populations of patients such as cancer patients or elderly people, to specific settings such as hospitals, hospices, or nursing homes, or is focused on a specific aspect of end-of-life care provision, thus providing only a limited view on how people are dying in a society [6-13]. Also, research exploring large-scale databases such as disease registries or healthcare billing data, or once-only population-based surveys studying quality end-of-life care is primarily restricted to the UK, Canada and US [14-17].

Furthermore, comparison of practices in different countries is often not feasible due to the use of different methodologies in different populations. One exception concerns the international analyses of mortality statistics based on official death certification, showing how many people die, at what age, from what causes, and where [6,13], but not including important other parameters of the quality of end-of-life care such as the use of palliative care, hospitalisations and transitions between settings, or communication at the end of life. Gathering such epidemiological data is pivotal to developing an effective public health policy on end-of-life care on a national and European level [5].

Because the general practitioner (GP) has a pivotal role in end-of-life care delivery in most countries in Europe – operating across care settings and patient populations – and because several countries within Europe have an operating Sentinel Network of General Practitioners – representative of all GPs in the country or region – the EURO SENTIMELC methodology offers unique possibilities to describe and compare end-of-life care in Europe on a continuous basis [18-21]. SENTIMELC refers to “Sentinel Network Monitoring End-of-Life Care”, an ongoing study which first started in Belgium in 2004 and in the Netherlands in 2005 and since 2009–2010 also involving Italy and Spain, focusing on describing and comparing end-of-life care in the last three months of life in different European countries – Belgium, the Netherlands, Italy and Spain in 2009–2010 – via the use of a representative network of GPs.

### Aims of the study

The objective of this report is to describe how representative GP networks in the EURO SENTIMELC study operate to monitor end-of-life care in a country, to describe used methodology and research procedures, and to present representativity and characteristics of the population reached using this methodology in 2009 and 2010. We hope that our experience in these 4 EU countries will be useful to others, in particular to countries with analogue surveillance networks of general practitioners who wish to integrate end-of-life care research into their registrations.

## Methods

Because of the problems with prognosticating who is dying in prospective end-of-life care research [22,23], we designed a continuous mortality follow-back study with data collection shortly after the patient had died, using a standardized registration form to be filled in weekly by the GP. The EURO SENTIMELC study was performed in 2009 and 2010 with data gathering in Belgium, the Netherlands, Italy and Spain (the latter limited to 2010 only). This European study is an expansion of the SENTIMELC study which first started in 2004 in Belgium and in the Netherlands in 2005 with continuing registrations since then. Several results for these two individual countries have been reported in previous publications [19,21,24-28]. In Table 1 an overview of all partners of EURO SENTIMELC is provided.

**Table 1:** EURO SENTIMELC consortium 2009-2010

	**Belgium – coordinator**	**The Netherlands**	**Italy**	**Spain**
Research institution	VUB-UGent End-of-Life Care Research Group, Vrije Universiteit Brussel	VU University Medical Center, EMGO Institute for Health and Care Research, Department of Public and Occupational Health	ISPO, Cancer Prevention and Research Institute	Directorate General of Public Health, Consejería de Sanidad, Valladolid
GP network	“Huisartsenpeilpraktijken”, the Belgian Sentinel Network of General Practitioners, coordinated by the Institute of Public Health, OD Public Health and Surveillance	“Continu Morbiditeitsregistratie Peilstations”, the Dutch Sentinel Network of General Practitioners, coordinated by the NIVEL Institute	Network from the Italian Society of General Practitioners	Health Sentinel Network of Castilla y León and the Health Sentinel Network of Comunitat Valenciana

### Observational unit

In this study, GPs are the observational units. Within Europe, general practice is highly accessible. GPs generally can provide a good public health perspective on end-of-life care and dying in the country. In some countries (e.g. the Netherlands, Spain), they are gatekeepers for healthcare delivery i.e. primary care providers who coordinate patient care and provide referrals to specialist services. In other countries (e.g. Belgium, Italy) they are not gatekeepers but do have a central coordinating role in the healthcare system with almost all of the population having a regular GP who they consult regularly. Hence, we use GPs in this study to generate a population-based sample of deaths. One important exception concerns the specialist nursing homes in the Netherlands since nursing home residents are treated by their own elderly care physicians and are thus outside of the view of the GP. Of all deaths in the Netherlands, 22% occurred in a nursing home in 2005 and average length of stay is more than one year.

In Belgium, the Netherlands and Spain *existing Sentinel Networks of General Practitioners* (GPs) are used as the observational unit in this study. A Sentinel Network of GPs is “a network of practices or community based physicians who monitor one, several or an exhaustive list of health problems on a regular or continuing basis”. The information from these practices is used to monitor the health of the entire population [18,29,30]. Recorded data must concern an important health problem not subject to surveillance of another system, unless the Sentinel Network provides complementary information to this end.

Given the existing Italian sentinel network mainly focuses on flu surveillance, the Italian partner has built its *own GP network* representative for the country and only performing registration regarding the end of life. To avoid selection of GPs more trained than the average GP in palliative care, Italian physicians were enrolled by the 9 coordinators of the participating health districts only specifying the procedure and not the content of the research. Participating physicians were sampled stratifying by age and sex, to assure representativeness of the GPs operating in the involved districts. Also, an extensive pilot phase was performed.

In Belgium, the GPs have been selected to cover the whole country and form a representative sample of GPs in the country. Each year, the responsible Institute for Public Health monitors the stability of the network (there is an annual turnover of 10% on average), verifies its representativity comparing age, gender and geographical distribution of the sentinel GPs with characteristics of the total GP population in Belgium, and calculates the percentages of the population coverage per district. Reports are published in in Dutch and French [31].

In the Netherlands (with an annual turnover of less than 5%), GPs are also selected to cover the whole country and each year the responsible institute verifies the representativity of the GP sample in terms of geographical distribution, urbanization, age and gender compared with the total GP population. Additionally, the sample of the population reached by the sentinel GPs is yearly compared in terms of age and gender with the whole patient population, to verify the representativity of the network – which is not possible for Belgium due to the lack of patient lists. These data are included in the yearly reports of the Dutch sentinel practices [32].

In Spain (with an annual turnover of less than 5%), the same procedures as in the Netherlands are performed within the two large regions in the Centre and East of Spain (Castilla y León and Valencia) to ensure representativity at GP and patient level. Spanish methodologies for setting up the sentinel networks have previously been published [30]. Sentinel GPs are randomly selected into clusters of the population to have the best representativity of the covered population. Age and sex distribution, as well as the socioeconomic status of the population is periodically compared to the general population of the regions [33-35].

In Italy, the nine health districts in the newly set up Sentinel Network of GPs are spread all over the country with three of them in large metropolitan cities (Genova, Palermo, Napoli). GPs were found representative in terms of age and gender in all 9 health districts participating (distributed in all of the four Italian statistical macro-areas). The distribution by age, sex and calendar period (month) of deaths registered by the Network was successfully compared with the last available national mortality statistics (2008).

All networks have in common that the participation of the GPs is voluntarily, and feedback is regularly distributed to the participants, concerned authorities, the medical press, scientific associations and interested individuals. The turnover of the GPs, from year to year, is low, which contributes to the collection of data of high scientific quality. Also, only regularly participating GPs (i.e. who register at least 26 weeks per year) are included for data analyses.

Further details on the participating GP networks can be found in Table 2.

**Table 2:** Characteristics of the participating GP networks in the EURO SENTIMELC study 2009-2010

	**Belgium**	**The Netherlands**	**Italy**	**Spain**
Coordinating institution	Institute of Public Health	NIVEL Institute	Italian Society of General Practitioners	Directorate General of Public Health
Founded in	1979	1970	2009	1988
Years of participation in EURO SENTIMELC study	Since 2004 and ongoing	Since 2005 and ongoing	2009 and 2010	2010
Participating regions	Country wide	Country wide	Country wide	Castilla y León (north) and Valencia (south)
Number of GP practices and general patient population coverage	+/− 200 GPs (+/− 170 GP practices) Covering 1.75% of the total Belgian population	+/−59 GPs (in 42 GP practices) Covering 0,8% of the total Dutch population	149 GPs participating in 2009; and 94 GPs in 2010 Covering +/− 3-4% of population per health district	+/−114 GPs covering 3,5% of the total +18y population in Castilla y León; and 59 GPs covering 2,2% in Valencia
Representativity of the GP network in the country	Representative of all GPs in Belgium in terms of age, gender and geographical distribution, and also of the GPs in the Northern (Dutch-speaking) and Southern (French-speaking) regions	Representative of all Dutch GPs in terms of geographical distribution and urbanization, age and gender	Representative of all GPs in terms of age and gender in all 9 health districts participating (distributed in all of the four Italian statistical macro-areas)	Representative for the 2 participating Sentinel GP Networks: Castilla y León and Valencia, in terms of age, gender, urbanization and other geographical variables

### Study population

The unit of measurement in the EURO SENTI-MELC study was the death case. Primary inclusion criteria were:

every patient, part of the practice of the GP, who had died (certified deaths and deaths of which they were informed afterwards)

aged 18 year or older

In order to focus this study on care delivered at the end of life or on dying patients (i.e. patients who were theoretically able to receive care in the terminal phase of life) we additionally excluded all deaths that had occurred “suddenly and totally unexpectedly” for some research questions [20,36,37].

### Retrospective data collection procedure

For the purpose of this study, the GPs registered deaths via a continuous and weekly standardized registration form, during 2 consecutive years (2009–2010) from January 1st until December 31^st^, except for Spain that joined the study in 2010. In Italy, GPs registered via a web based electronic questionnaire while in all other countries GPs used paper and pencil (expect for Valencia that uses electronic registering). To shorten the time between death and registration – hence preventing recall bias as much as possible – the physicians were instructed to register all deaths, immediately after being informed about the patient’s death. GPs use patient records and information coming from hospital physicians as much as possible when filling in the forms. GPs are sent accompanying instructions at the beginning of each year, clearly stating the inclusion criteria of the study and clarifying the manner in which some questions need to be filled in. The specific operating procedures that are used by an existing Sentinel Network are also followed for the end-of-life care registration. Table 3 provides details on the data collection procedures in each country.

**Table 3:** Data collection procedures of the participating GP networks in the EURO SENTIMELC study 2009-2010

**Data collection procedures**	**Belgium**	**The Netherlands**	**Italy**	**Spain**
Frequency and mode of reporting	Weekly reporting Paper and pencil	Weekly reporting Paper and pencil	Weekly reporting online web based registration (Emailing with memo sent weekly)	No weekly reporting: deaths are reported the week of the event; however GPs are used to send in a weekly report form on other health problems
				Paper and pencil for Castilly y Léon; electronic registry for Valencia
Extra quality control measures	-selection of regular participating GPs (registered 26 weeks or more of one year)	-selection of regular participating GPs (registered 26 weeks or more of one year)	-it concerned a new network only involving GPs that agreed to participate for a whole year	-selection of regular participating GPs (registered 26 weeks or more of one year)
	-data entry by the Institute of Public Health using dbase-based programme to prevent key punching errors, double data entry by VUB	-data entry by researchers, 5% with double data entry	-web based application needing no data entry and ensuring all necessary items are filled in	-data entry by province coordinators, using dbase-based programme to prevent key punching errors; no double data entry
	-automatic follow-up forms to prevent missing data for key variables; telephone contact with GP also possible	-reminders send by NIVEL after checking for missing data on key variables; if necessary telephone contact with GP	-weekly reminders (an e-mail with a memo was sent weekly to assure the ready reporting of deceased cases)	-reminders to GPs when missing data or inconsistencies
			
	-GPs received summaries of all reported deaths after each year of registration (2005–2006 to verify for possible non-response (e.g. GPs who forgot to report one of their deaths)	-GPs have patient lists	-GPs have patient lists	-GPs have patient lists
Anonymity procedures	-anonimization of patient data upon data entry	-anonimization of patient data upon data entry	-anonimization of patient data when registering	-anonimization of patient data upon data entry in Castilla and after data recording in Valencia
	-anonimization of physician data when closing database	-anonimization of physician data when closing database	-anonimization of physician data when closing database	-anonimization of physician data when closing database
Training for GPs	-registration instructions each year	-registration instructions each year	-registration instructions via coordinating GPs per health district at the beginning of the year	-registration instructions each year
	-yearly individualized feedback on basic parameters	-yearly presenting of results on meeting of participating GPs		-yearly individualized feedback on basic parameters

### Definition of concepts

In this study, we mainly focus on the final three months of life of patients and investigate several important components of quality of end-of-life care: places of care and death, transitions between care settings; communication; palliative care provision; symptoms in the last week of life; and costs/burden of end-of-life care. These domains have been identified in international literature as important components of quality of end-of-life care[15,16,38-40], and are particularly relevant from the GP perspective not being under surveillance via other instruments.

*Transitions between end-of-life care settings* were defined as moves or changes in location of care during the last three months of life. Home (or with relatives, in service flats), care home (including homes for elderly people in all four countries and nursing homes in Belgium, Italy and Spain excluding the specialist Dutch nursing homes), hospital and inpatient palliative care unit, were differentiated.

Concerning *communication* we differentiated between:

topics addressed during *conversations* between GP and the patient

elements of *advance care planning*:

• preferences for place of death

• wishes about a medical treatment s/he would or would not want in the final phase of life

• wish for a proxy decision-maker

*Palliative care* was studied in terms of palliative care delivered by the GP and *specialist multidisciplinary* palliative care [1]. As there are differences in the types of multidisciplinary palliative care services offered in each country, each service was classified into one of three categories in order to facilitate comparison, as shown in Table 4.

**Table 4:** Specialist multidisciplinary palliative care services in the four participating countries of the EURO SENTIMELC study

	**Belgium**	**Netherlands**	**Italy**	**Spain**
Hospice/palliative care unit	Palliative care unit in a hospital	Hospice, palliative care unit (in a hospital, nursing home, or care home)	Hospice	Palliative care unit in a hospital
Palliative care service for patients staying at home	Palliative home care team, palliative day care centre	Palliative care consultation team^*^	Palliative home care team, domiciliary integrated assistance with palliative care	Palliative home care team, palliative day care centre, ambulatory palliative care in a hospital
GP with palliative care training	^§^	GP with palliative care training†	^§^	^§^
In-house palliative care service in a nursing home (excl. The Netherlands)	Reference persons for palliative care in a nursing home	^‖^	^‖^	Palliative care nurses in a nursing home
Hospital-based palliative care service (excl. palliative care unit)^‡^	Mobile palliative care support team in a hospital	Palliative care consultation team^*^	Pain therapy or palliative care specialist consultation during a hospital admission	^‖^

^*^ Palliative care consultation teams mainly offer services to patients at home but also to patients in hospital/hospice/nursing home.

^**†**^ GPs followed palliative care training offered by the Dutch Association of General Practitioners (Nederlands Huisartsen Genootschap, NHG); they are registered as palliative care advisors in a central database.

^**‡**^ For patients admitted to hospital for at least one day in the last three months of life.

^**§**^ Not available/assessed in this country.

^‖^ Not available in this country.

### Measurement instrument

A majority of the items are pooled from existing registration forms used in the SENTIMELC study in Belgium and the Netherlands. These were developed on the basis of previous retrospective and quantitative studies whenever possible [6,15,20,37,41,42].

In case a specific concept could not be measured with an existing instrument, questions were developed on the basis of relevant literature and in dialogue with the all partners (the GP Networks and Researchers) and a Belgian/Dutch Advisory Board consisting of GPs, palliative care physicians, psychologists, nurses, medical sociologists, health scientists and an anthropologist. Also, new questions were tested among GPs before using them. Based on our previous experiences with GP registrations, we mainly used structured and closed-ended questions.

Questions were developed in Dutch and translated into French and English via forward-backward procedures. Italian and Spanish versions were developed from the English version via the same procedures. Translations were performed by independent native-speaking persons and discrepancies discussed and deliberated. The translation allowed for cultural differences in words or health care structures (eg differences in places of care, palliative care services). The registration form is shown in Additional file 1 in English.

### Feasibility testing

An extensive pilot study to test the feasibility of the study design and the measurement instrument was performed in Belgium in 2004 [43]. For the EURO SENTIMELC study, the questionnaire was pretested in all countries using the following procedures:

a. 10–15 GPs (preferably sentinel GPs) per country were asked to fill in the registration form for pretesting purposes, in a face-to-face interview situation.

b. GPs were asked to fill in two registration forms: one for the most recent sudden and totally unexpected death case in their GP practice; and one for the most recent non-sudden or expected death case in their GP practice.

c. The physician filled in the registration form independently and without any help.

d. All physicians were asked to report any problem (out loud) that was encountered while filling in the registration form, concerning eg clarity of the questions, instructions for filling it in, difficulty of providing the requested information etc.). The time that was required to fill it in was also noted.

All issues were collected by the coordinator who made final decisions after consulting the partners.

### Ethical considerations

The participating GPs were asked to give written informed consent at the beginning of a registration year, after being fully informed about the objectives and method of the research themes. If an existing GP network was used, the standard operating procedures within the Network were used. Additionally, strict procedures regarding patient anonymity are employed. Every patient that was registered within the network receives an anonymous reference from the GP him/herself. There also was supplementary coding of patient information i.e. the patient’s date of birth might be registered by the GP but was replaced with the patient’s age before data-entry, and postal code of habitual residence was transformed into more aggregate indicators such as province and region of care. Concerning the GPs’ identity, all his/her identification codes were replaced in the data files with anonymous codes during data cleaning in each country.

The protocol of the present study was approved by the Ethical Review Board of Brussels University Hospital of the Vrije Universiteit Brussel and the Local Ethical Committee ‘Comitato Etico della Azienda U.S.L. n. 9 di Grosseto’,, Tuscany. According to specific regulations, no specific ethical approvals were needed in the Netherlands or Spain because of the retrospective and anonymous data collection.

### Data management and statistical analysis

Each GP network had its own control measures to ensure data quality and to limit missing data (see Table 3 for details). A common format for coding of variables, used by all partners, was made by the coordinator, who also merged all files. All operations were stored via SPPS syntax-files.

To verify if a representative population-based sample of deaths can be obtained via this representative sample of GPs, we evaluated whether the deaths registered by the Sentinel Network of GPs are comparable in terms of age, sex and place of death to the deaths occurring within the general population or to the characteristics of the non-sudden deaths within the population (if possible).

## Results

The number of participating GPs and their population coverage per country in 2009 was 199 (1.8%) in Belgium, 59 (0.8%) in the Netherlands, and 149 (4.3%) in Italy. The respective figures for 2010 were 189 (1.5%) in Belgium, 63 (0.8%) in the Netherlands, 94 (2.7%) in Italy, and 173 in Spain (114 (3.4%) in Castilla and León, 59 (3%) in Valencia).

Table 5 shows the number and characteristics of the registered deaths in the EURO SENTIMELC study of 2009–2010 and of the percentages of nonsudden deaths in each country. A total of 6858 deaths were registered of which two thirds died non-suddenly (from 62% in the Netherlands to 69% in Spain). Of these non-sudden deaths between 32% and 44% of deaths were aged 85 or older; between 46% and 54% were female, and between 23% and 49% died at home. Cancer was cause of death in 37% to 53% of non-sudden death cases.

**Table 5:** Number and characteristics of reported deaths in the EURO SENTIMELC study 2009–2010

		**Belgium**		The Netherlands*		**Italy**		**Spain**†	
		**All deaths**	**Non-sudden deaths§**	**All deaths**	**Non-sudden deaths§**	**All deaths**	**Non-sudden deaths§**	**All deaths**	**Non-sudden deaths §**
		**N = 2405**	**N = 1604 (66.7%)**	**N = 1107**	**N = 635 (62.1%)**	**N = 2783**	**N = 1839 (66.1%)**	**N = 563**	**N = 388 (68.9%)**
		**%**	**%**	**%**	**%**†	**%**	**%**	**%**	**%**
Age	18-64	16	14	21	18	13	13	11	11
	65-84	47	47	50	50	48	47	46	45
	85+	37	39	29	32	39	40	43	44
Gender	Male	48	46	52	47	48	47	53	54
	Female	52	54	48	53	52	53	47	46
Place of death‡	Home	29	23	45	44	48	46	48	49
	Care or residential home	27	31	14	18	7	9	11	13
	Hospital	37	36	32	28	39	39	37	33
	PCU/hospice	7	9	7	10	4	5	4	4
Cause of death	Cancer	28	37	37	53	34	46	35	39
	Noncancer	72	63	63	47	66	54	65	61

*Excluding nursing home deaths in the Netherlands.

† Data for Spain are available only for the year 2010.

‡IT and SP: each 1% POD elsewhere.

§% missing on nonsudden deaths : 0.7% for Be, 0% for the Ne, 1.7% for It and 5.9% for SP.

Percentages are rounded off hence cells may not add up to 100%.

Table 6 also compares the characteristics of the registered sample with the characteristics of a reference population. In Belgium and the Netherlands, figures for all non-sudden deaths are compared with a previous death certificate study on end-of-life decisions (in 2007 for Belgium and 2005 for the Netherlands) in which a representative sample of non-sudden deaths was obtained after weighting [37,44]. For Italy and Spain, figures for all deaths are compared with the total death rates in the country or in the reference population. Overall we find no large differences between the obtained SENTIMELC samples and the reference populations. In all countries, GPs can identify deaths due to cancer and noncancer, dying at home as well as in institutional settings. We do see a slight underrepresentation of non-sudden hospital deaths and people under the age of 65 in Belgium and a slight underrepresentation of females in the Netherlands. We assume that GPs in Spain and Italy might also miss out on some of the sudden deaths occurring in the hospitals, but cannot test this hypothesis due to the absence of place of death information on death certificates.

**Table 6:** Representativitity of reported deaths in the EURO SENTIMELC study 2009–2010

		**Belgium**			**The Netherlands**			**Italy**			**Spain**		
		**Non-sudden deaths in the SENTI-MELC study**	**Non-sudden deaths in the 2007 death certificate study**		**Non-sudden deaths in the SENTI-MELC study**	**Non-sudden deaths in the 2005 death certificate study**		**All deaths in the SENTI-MELC study**	**All deaths in the country 2007**		**All deaths in the SENTI-MELC study**	**All deaths in the regions 2010**	
		%*	%*	p**	**%**†	**%**†	p**	**%**	**%**	p**	**%**	**%**	P**
		**N = 928**	**N = 2729**		**N = 635**	**N = 63038**		**N = 2783**	**N = 572881**		**N = 388**	**N = 67233**	
Age	1-64	13	16	0.01	18	21	ns	13	14	ns	11	15	ns
	65-84	50	52		50	36		48	50		46	46	
	85+	37	32		32	43		39	36		43	39	
Gender	Male	46	47	ns	47	51	.04	48	49	ns	53	52	ns
	Female	54	53		53	49		52	51		47	48	
Place of death§	Home	23	21	.0001	44	33	ns	48	‡	-	48	‡	-
	Residential home	31	25		18	16		7	‡		11	‡	
	Hospital PCU/hospice	45	51		38	47		43	‡		41	‡	

* Numbers for Flanders, the Dutch-speaking part of the country (60% of the population) only, due to absence of death certificates database in the French-speaking part of Belgium.

† Excluding nursing home deaths in the Netherlands.

‡ Unknown for Italy and Spain. Although data on place of death are not available in Italy from the national official statistics, the results for cancer deaths were similar to the ISDOC study figures of 2003 [45].

§ IT and SP: each 1% POD elsewhere; BE 3% other.

**p-values using Fisher exact test.

## Discussion

The objectives of the EURO SENTIMELC study are to describe end-of-life care provided to patients who had died non-suddenly in Belgium, the Netherlands, Italy and Spain and to make cross-country comparisons. We also aim to investigate patient, disease and healthcare characteristics associated with variations in end-of-life care. To realize these objectives, this retrospective study with data collection via four representative Networks of General Practitioners was set up.

### Interpretation of the main findings

The need for setting up standardized continuous systems to monitor where and how people die has been recognized worldwide [1,3-5]. The lack of population-based and nationwide studies on end- of-life care hampers the ability to develop an effective public health policy on end-of-life care and has been identified as a major gap in end-of-life research today. Also, improving clinical practice starts with good monitoring of the current situation [1,3-5].

Death certificate data or mortality statistics are by far the most known public health method used to inform where, at what age and due to what cause deaths occur in a country [6,46]. However, the resulting data are very limited in scope eg they do not included data on the circumstances of dying and place of death is coded in a limited way (eg in most countries palliative care units cannot be differentiated from hospital deaths and in some other countries hospital deaths can only be distinguished from deaths in ‘other places’) [46]. Several efforts have been made to explore data relevant for end-of-life care in existing large-scale databases such as disease registries, hospital discharge registers, disease-specific registers or healthcare billing data [7,16,17,47]. However, no data source is totally comprehensive and all have their specific limitations on a national and international comparative level. Tracking patients, cancer and non-cancer, throughout the health care systems – transferring between health care settings– has been found to be particularly challenging in end-of-life care research today [1,4,16]. Hence, using primary care GP networks that register data which is not systematically gathered via other data collection systems is of considerable added value for monitoring death and dying from a societal point of view, comparing countries and providing a good basis for continuous surveillance. Our results show that representative samples of GPs in the four countries involved have the potential of providing a good public health perspective on end-of-life care and dying in a country.

This study has several potential strengths as well as limitations associated with the use of an (existing) surveillance network of GPs in general, and with the specific design of the study.

### Strengths

The Sentinel Network of General Practitioners in Belgium, the Netherlands and Spain are representative of all GPs in the country or region, have a long tradition in scientific research, are flexible in terms of acceptability of new registrations, and very stable in terms of participating GPs. The GPs are motivated to monitor various health-related problems over long and repeated periods, but have not been selected on the basis of a specific interest in end-of-life research. In Italy, where a new network has been set up, GPs were not informed about the content of the research beforehand to avoid selection of GPs more trained than the average GP in palliative care. They were selected to be representative for 9 large health districts distributed all over the country. However, the GPs that are motivated to participate in these type of registration networks, might still represent a selective group of physicians interested in scientific research, a limitation that is probably also present in other studies.

Because detailed information concerning the care provided is not always available from the patients’ medical files, nor from existing deaths registers such as death certificate data, a registration directly with GPs has important surplus value. Using the representative networks, we could obtain samples of deaths representative for the GP populations in the participating countries. A specific strength of this retrospective study is that memory bias found in other retrospective designs [22,36], will be limited, because of the weekly registrations, leaving little time between death and registration. Also, identification of non-sudden deaths as denominator is an advantage over other prospective and retrospective designs that have been criticized for selecting patients solely on the basis of diagnose or cause of death. Not all patients with a cancer diagnose, for example, also die of cancer or receive care with an end-of-life intent [23,48]. By avoiding including patients that died suddenly and unexpectedly, we will be able to study care that was truly delivered in the context of a dying process. Additionally, while retrospective designs may have their limitations to resurrect certain aspects of the treatment histories of deceased patients [48], it is the most appropriate design to identify a representative sample of deaths and to make population-based estimates about who received palliative care [49,50]. Prospective follow-up studies cannot follow all patients until death hence leaving patients living the longest underrepresented [23,51]. Another strength concerns the possibility of making cross-country comparisons in populations attended by GPs and in the care provided at the end of life, using analogous methodologies. Finally, many different end-of-life topics can be studied via GPs.

### Weaknesses

The registration form is to be kept simple, and time-consuming questions should be avoided in a surveillance system. Consequently, in-depth study of some aspects of care is generally not possible via this type of registration research and observed cross-country differences might be difficult to explain [18]. Possible weaknesses also include the retrospective data collection approach making reconstruction of all care provided in the final three months of life difficult [22] and the reliance on GPs to report care and decisions at the end of life, including care delivered to patients in hospitals or decisions taken by hospital physicians. An underestimation of specific types of care provided or decisions taken is thus possible. A specific weakness for Belgium is that there are no patient lists per practice, hence the population denominator (the “sentinel population”) is not precisely defined and has to be estimated on the basis of annual total number of patient encounters in the participating practices [52]. Additionally, while using the same methodology in the different countries, the population of dying patients that is taken care of by the GP differs per country – e.g. we lack nursing home deaths in the Netherlands – which makes it necessary to correct for these differences when comparing countries. Finally, GPs appear to underreport a limited number of deaths ie non-sudden hospital deaths and deaths of people under 65 years old in Belgium, and possibly also sudden hospital deaths in all countries.

### Opportunities for further research

Because data can be gathered over time, we will, in the long-run, evaluate the monitoring potential of this instrument. The results could potentially serve as baseline data to monitor end-of-life care over time. Also, since many other European countries have at least one Sentinel Network of GPs [18,29], the study provides opportunities for further comparisons with other countries. The nationwide Sentinel Network from France has expressed interest and other countries are being contacted to join this monitoring study in 2013. Finally, the EURO SENTIMELC database is made available for the EURO IMPACT project, a EU funded Marie Curie Initial Training Network aimed at describing quality of end-of-life care for cancer and noncancer patients and identifying tools to improve it (http://www.euro-impact.eu) that is researchers will analyse and publish the obtained data. This creates important opportunities for large scale dissemination of the results nationally and internationally.

## Conclusions

In the EURO SENTI-MELC study, we will build a public health database on how people are dying and what care they are receiving at the end of life in different countries in Europe and make cross-country comparisons. It will provide important information for practitioners and healthcare policy makers which they can use to determine their future priorities.

## Abbreviations

SENTI-MELC: Sentinel network Monitoring End-of-Life Care; GP: General practitioner.

## Competing interests

The authors declare that they have no competing interests.

## Authors' contributions

LVDB, BOP, GD, GM, VVC, TVA and LD were involved in the conception and design of the study. LVDB, KM, GD, FG, VVC, TVA, and OZ gathered the data. Statistical analyses are carried out by LVDB. The manuscript was drafted by LVDB with critical input from all other authors. LVDB was the project supervisor. All authors read, revised and approved the final manuscript.

## Pre-publication history

The pre-publication history for this paper can be accessed here:

http://www.biomedcentral.com/1471-2296/14/73/prepub

## Supplementary Material

Additional file 1 Registration form of EURO SENTIMELC study 2009–2010 file name deces 2010 ENG file format. Click here for file
