# Supplementary material for: Nationwide continuous monitoring of end-of-life care via representative networks of general practitioners in Europe
Source: BMC Fam Pract. 2013 Jun 3;14:73. doi: 10.1186/1471-2296-14-73 (PMC3751186; doi:10.1186/1471-2296-14-73)
Supplement: Additional file 1 — Registration form of EURO SENTIMELC study 2009–2010 file name deces 2010 ENG file format. [file 1471-2296-14-73-S1.docx]

End-of-life care in Europe

**a descriptive mortality follow-back study via representative Networks of General Practitioners**

**The standardized weekly registration form of the EURO SENTI-MELC study 2010**

**© The EURO SENTIMELC RESEARCH GROUP 2010**

**BELGIUM**

Research Partner End-of-Life Care Research Group, Vrije Universiteit Brussel

In collaboration with the Huisartsenpeilpraktijken i.e. the Belgian Sentinel Network of General Practitioners, coordinated by the Institute of Public Health, Department of Epidemiology

**THE NETHERLANDS**

Research Partner VU University Medical Center, and EMGO Institute

In collaboration with the Huisartsenpeilstations, the Dutch Sentinel Network of General Practitioners, coordinated by the NIVEL Institute

**ITALY**

Research Partner ISPO, University of Florence

In collaboration with the Italian Society for General Medicine

**SPAIN**

Sentinel Network of General Practitioners of Junta de Castilla y León, co-ordinated by Consejería de Sanidad

Please contact the EURO SENTIMELC Research Group Coordination if you want to use this instrument, in full or individual items,

or if you want to adapt it to your local circumstances:

Contact address:

Vrije Universiteit Brussel, End-of-Life Care Research Group Laarbeeklaan 103 - 1090 Brussels, Belgium

Tel +32 2 477 43 10 - Fax +32 2 477 47 11 - [www.endoflifecare.be/ZrL](http://www.endoflifecare.be/ZrL) [lvdblock@vub.ac.be](mailto:lvdblock@vub.ac.be)

**REGISTRATION OF ALL DEATHS OF PATIENTS (aged 1 year or older) WHO ARE PART OF YOUR (GROUP) PRACTICE**

1. Your reference (e.g. initials) : ......................................................

2. Date of birth : . . / . . / . . . . 3. Date of death : . . / . . / 2 0 1 0

4. Gender : M F 5. Postal code of patient’s usual place of residence

|  |  |  |  |
| --- | --- | --- | --- |

1. Where did the patient reside the longest in his/her last year of life?

at home or living with family care home(nursing home/home for elderly persons) other (namely) .......................................

1. Cause of death*: lllness or disorder that was the direct cause of death: State below under (a) the logical association of the illnesses/disorders that resulted in the immediate cause of death. If more than one illness, state the illness that was the "original cause of death" last. ***This is not the way in which the patient died, e.g. heart failure, syncope, etc. ... but the illness, the trauma or the complication that caused the death.** Please mention only one cause per line.

(a) ............................................................................................................................................................................................

Caused by : (b) ......................................................................................................................................................................

Caused by : (c) ......................................................................................................................................................................

Caused by : (d) ......................................................................................................................................................................

see instructions for examples

1. Did you or another doctor determine the diagnosis of dementia ? yes, severe dementia yes, mild dementia no unknown

9a. The **place of death** of the patient and **place(s) of residence** during the **last 3 months (=90 days) before death**, as well as the duration of stay in **days** (approximately if not precisely known). If the patient remained in the same place until death, only fill in Place 1.

at home or care home : hospital (**excl.** palliative elsewhere

see instructions

for examples

living with home for the pall. care unit, care unit **please specify**

family(incl. elderly / and **excl.** nursing

service flat) nursing home home unit in hospital

- 1. place of **déath** and . . . . days . . . . days . . . . days . . . . days . . . . . . . . . . . . . duration of stay . . . . days
  2. place of **déath** and . . . . days . . . . days . . . . days . . . . days . . . . . . . . . . . . . duration of stay . . . . days
  3. place of **déath** and . . . . days . . . . days . . . . days . . . . days . . . . . . . . . . . . . duration of stay . . . . days
  4. place of **déath** and . . . . days . . . . days . . . . days . . . . days . . . . . . . . . . . . . duration of stay . . . . days

9b. To be filled in ONLY IF THE PATIENT WAS MOVED one or more times during the final three months of life: for what reason(s) was the patient

**moved to the place where s/he died**? (*More than one answer can be given)*

it was the wish of the patient

it was the wish of the patient's family or significant other(s)

the patient needed (more) palliative care or palliative treatment(s) the patient needed curative or life-prolonging treatment(s)

the patient did not need further treatment in that setting

other (please specify) : .................................................................................................................................................................................

10. How often, on average, did you have **contact** (consultations, home visits, excl. telephone contact) with the patient or with significant others regarding the patient?

| **last week before death** | **2nd to 4th week before death** | **2nd and 3rd month before death** |
| --- | --- | --- |
| . . . x per **week** | . . . x per **week** | . . . x per **month** |

| 11. | Were the following topics addressed during **your conversations with the patient**?  *(1 answer per line)* | yes | no | not  applicable | | |
| --- | --- | --- | --- | --- | --- | --- |
| A primary diagnosis ------------------------------------------------------------------------------------------------------------- | | ---------- |  | ---------- |  | ----------- |
| B incurability of the illness ---------------------------------------------------------------------------------------------------- | | ---------- |  | ---------- |  | ----------- |
| C life expectation ---------------------------------------------------------------------------------------------------------------- | | ---------- |  | ---------- |  | ----------- |
| D possible medical complications ------------------------------------------------------------------------------------------- | | ---------- |  | ---------- |  | ----------- |
| E physical complaints ---------------------------------------------------------------------------------------------------------- | | ---------- |  | ---------- |  | ----------- |
| F psychological problems (i.e., sadness, worry, fear) --------------------------------------------------------------  G social problems (i.e., relationship problems, lack of social support from family/friends,  family unable to accept situation, etc.) --------------------------------------------------------------------------------- | | ----------  ---------- |  | ----------  ---------- |  | -----------  ----------- |
|  | H spiritual/existential problems (i.e., difficulty in accepting the situation, trouble with the  meaning of life, angry at God, etc.) ------------------------------------------------------------------------------------- | ---------- |  | ---------- |  | ----------- |
|  | I options for palliative care -------------------------------------------------------------------------------------------------- | ---------- |  | ---------- |  | ----------- |
|  | J burden of treatments -------------------------------------------------------------------------------------------------------- | ---------- |  | ---------- |  | ----------- |
| 12. | How **important** do you think the following aspects were in the care of this patient? |  |  |  |  |  |

**1**=not at all important **5**=very important **?**=unknown

last week before death 2nd to 4th week before death 2nd and 3rd month before death

| treatment aimed at **cure --------------------------------** | 1 | 2 | 3 | 4 | 5 | ? | 1 | 2 | 3 | 4 | 5 | ? | 1 | 2 | 3 | 4 | 5 | ? |
| --- | --- | --- | --- | --- | --- | --- | --- | --- | --- | --- | --- | --- | --- | --- | --- | --- | --- | --- |
| treatment aimed at **prolonging life ---------------** | 1 | 2 | 3 | 4 | 5 | ? | 1 | 2 | 3 | 4 | 5 | ? | 1 | 2 | 3 | 4 | 5 | ? |
| treatment aimed at **comfort/palliation -----------** | 1 | 2 | 3 | 4 | 5 | ? | 1 | 2 | 3 | 4 | 5 | ? | 1 | 2 | 3 | 4 | 5 | ? |

13. Within the **last week** of life, was the patient capable of making decisions? yes sometimes no unknown

14a. Did **you** provide palliative care to this patient? yes, until death yes, but not until death no

14b. Which **palliative care initiatives** were involved in the last 3 months of this patient's life?

*(More than one answer can be given)*

palliative homecare team

mobile palliative care support team in a hospital palliative care unit (hospital)

reference persons* for palliative care in a care home palliative day care centre

other (namely) : ........................................................................

none

**14c. Estimate the number of days between the first palliative intervention and the moment of death ...... days**

unknown *(coordinating and advisory physician and/or reference nurse)

1. How difficult was it for the patient and his/her family to cover the **costs** of the care in the last three months of the patient's life? very difficult somewhat difficult not difficult at all don’t know patient did not need care
2. Did the informal caregivers feel **overburdened** (physically or emotionally) in the last three months of the patient's life? yes no don’t know there were no informal caregivers
3. Were you informed (verbally or in writing) of the patient's preference regarding place of death? *(More than one answer can be given)*

YES by the patient him/herself

by the patient's family or significant other

other (namely) : ...............................................................................................................................................................................

If **YES**, where did this patient prefer to die? at home or living with family (incl. service flat)

in a care home

in hospital (excl. palliative care unit, and excl. nursing home unit in hospital) palliative care unit (hospital)

elsewhere (namely) : ........................................................................................

¡ NO

1. Did the patient ever express specific **wishes** about a medical treatment that he/she would or would not want in the final phase of life? yes no unknown

If **YES,**

- 1. Did you ever speak to the patient about these wishes? yes no
  2. Within the last week of life, was there any medical procedure or treatment that was inconsistent with previously stated wishes? yes no unknown

1. Did the patient ever express a **wish** about who was to make decisions regarding medical treatments or activities **in his/her place**, **in the event he/she would no longer be able to speak for him/herself**? *(More than one answer can be given)*

yes, in writing yes, verbally no unknown

If **YES,**

- 1. Did **you** ever speak to the patient about these wishes? yes no
  2. If the situation did arise, was this person consulted? yes no unknown situation did not arise

1. Did the patient have the following symptoms during the last week prior to death?

*(Please circle the most appropriate answer)* If **YES,** how much did that **distress** the patient?

**Yes No Unknown** not at all a little bit somewhat quite a bit very much unknown

| A lack of appetite ---------------- | --- | ------- | ------------------- 1 ---------------- 2 ----------------- | 3 ------------ | 4 ---------- | 5 ---- | ----- |
| --- | --- | --- | --- | --- | --- | --- | --- |
| B lack of energy ------------------ | --- | ------- | ------------------- 1 ---------------- 2 ----------------- | 3 ------------ | 4 ---------- | 5 ---- | ----- |
| C pain -------------------------------- | --- | ------- | ------------------- 1 ---------------- 2 ----------------- | 3 ------------ | 4 ---------- | 5 ---- | ----- |
| D feeling drowsy ---------------- | --- | ------- | ------------------- 1 ---------------- 2 ----------------- | 3 ------------ | 4 ---------- | 5 ---- | ----- |
| E constipation --------------------- | --- | ------- | ------------------- 1 ---------------- 2 ----------------- | 3 ------------ | 4 ---------- | 5 ---- | ----- |
| F dry mouth ----------------------- | --- | ------- | ------------------- 1 ---------------- 2 ----------------- | 3 ------------ | 4 ---------- | 5 ---- | ----- |
| G difficulty breathing ------------ | --- | ------- | ------------------- 1 ---------------- 2 ----------------- | 3 ------------ | 4 ---------- | 5 ---- | ----- |

If YES, how **often** did the patient appear to feel this way?

rarely occasionally frequently almost unknown

constantly

H feeling sad ---------------------- --- ------- -------------------- 1 --------------- 2 ------------------- 3 --------------- 4 -------- ---

I worrying ------------------------- --- ------- -------------------- 1 --------------- 2 ------------------- 3 --------------- 4 -------- ---

J feeling irritable ----------------- --- ------- -------------------- 1 --------------- 2 ------------------- 3 --------------- 4 -------- ---

K feeling nervous ---------------- --- ------- -------------------- 1 --------------- 2 ------------------- 3 --------------- 4 -------- ---

1. **Was death sudden and totally unexpected?** yes no
2. Was the patient suffering from a life-threatening illness or condition (incl. coma) OR from serious health problems associated with old age? yes no
